# Mechanism, application and effect evaluation of nerve mobilization in the treatment of low back pain: A narrative review

**DOI:** 10.1097/MD.0000000000034961

**Published:** 2023-08-25

**Authors:** Quanzheng Chen, Zhenshan Wang, Shuna Zhang

**Affiliations:** a Department of Physical Education and Health, Guangxi Normal University, Guilin, China.

**Keywords:** low back pain, lumbar spine, manual therapy, nerve mobilization, physical therapy

## Abstract

Lower back pain is a prevalent condition affecting people across all age groups and causing significant personal and societal burdens. While numerous treatments exist, nerve mobilization has emerged as a promising approach for managing lower back pain. Nerve mobilization involves applying gentle and rhythmic movements to the affected nerves, promoting normal nerve function and releasing tension. It has been well documented that nerve mobilization can be effective in reducing pain and improving function in patients with lower back pain, but the underlying mechanisms have not been clarified. This study aims to review the mechanisms of nerve mobilization in the management of lower back pain, its application, and effectiveness evaluation, and provide a potential solution for managing lower back pain.

## 1. Introduction

Low back pain (LBP) refers to the pain or discomfort experienced in the region of the back between the lower back and the sacrum.^[1]^ This condition is classified as a musculoskeletal system problem that can be caused by injury or damage to the spine, muscles, and ligaments,^[2]^ the overwhelming majority of individuals will experience LBP at some point in their lifetime.^[3,4]^ According to statistical data, LBP is a highly prevalent condition worldwide, with more than 80% of individuals experiencing it at some point in their lives. Furthermore, it is a significant cause of disability and can impact an individual’s work status.^[5,6]^ Lower back pain can cause pain, stiffness, muscle spasms, and reduced mobility, all of which can significantly impact an individual’s health and overall quality of life.^[7,8]^ Furthermore, lower back pain can also have negative effects on an individual’s emotional and mental well-being, leading to feelings of anxiety, depression, and other negative emotions.^[9]^ As the global population continues to grow and the problem of aging becomes more prevalent, the burden of chronic and persistent LBP will also increase. This highlights the urgent need for more effective treatment strategies to manage LBP, which is traditionally treated with medication such as nonsteroidal^[10]^ anti-inflammatory drugs, muscle relaxants, and opioids^[11]^; Traditional physiotherapy techniques for managing LBP include massage therapy, chiropractic care, as well as strength and flexibility exercises^[12]^; Surgical interventions may be considered for patients with higher levels of severity of LBP.^[13]^ Although all of these treatment options can be effective in managing pain, they also have their shortcomings. Medication can have certain side effects, and the regular use of opioids for pain relief may lead to drug dependence^[14]^; Short duration of pain control, low economic efficacy, and other limitations are associated with traditional physiotherapy techniques.^[15]^ Nerve mobilization (NM) is a physiotherapy technique performed by a specialist physiotherapist that involves moving, pulling, or vibrating nerve tissue to improve the function of the nervous system. This technique is primarily used to treat neuropathy, nerve pain, muscle tension, and other neurological problems.^[16,17]^ Some study has assumed that NM is widely regarded as one of the most effective treatments for accelerating pain relief and promoting recovery. This technique achieves these effects by promoting blood and lymphatic circulation, tissue repair and regeneration, and modulating nerve activity in various ways.^[18]^ NM is a noninvasive and targeted treatment approach that differs from surgery and conventional treatments. It involves mobilizing a specific nerve using a variety of techniques and mechanisms to reduce pain, restore normal nerve function, and improve LBP status.^[19]^ NM has been shown to produce sustained results, making it a promising treatment option for individuals with LBP. Despite its potential benefits as a noninvasive treatment for lower back pain, the specific factors and mechanisms influencing the effectiveness of NM are not yet fully understood. This paper aims to provide more accurate and comprehensive recommendations and protocols for the management of lower back pain by offering a systematic summary of the mechanisms of action of NM in treating LBP, its application, and the evaluation of its effectiveness.

## 2. Mechanism of action of nerve mobilization in the treatment of low back pain

### 2.1. Nerve regeneration and recovery

LBP can lead to structural changes in the spine, which may increase the risk of nerve root compression. Compression or injury to the nerve roots can cause a range of symptoms, such as numbness, pain, weakness in the lower extremities, and slowed nerve conduction.^[20,21]^ After nerve damage, there is an alteration in neuronal metabolism, leading to an acceleration of protein synthesis. This process promotes the regeneration of nerve growth factor and brain-derived neurotrophic factor, ultimately speeding up the recovery of nerve function, including sensory and motor functions in humans.^[22]^ In an animal experiment, it was found that NM has been found to enhance nerve activity, stimulate nerve fibers, promote nerve regeneration, and increase nerve conduction velocity through targeted traction or compression of specific nerves.^[23]^ Studies have also demonstrated that NM can promote cell growth in sensory and motor neurons through manipulation patterns in vitro at different doses, and can have selective antiapoptotic effects in sensory neurons, which further aids in promoting cell recovery.^[24]^ Currently, axonal regeneration is considered the primary mechanism of nerve repair.^[25]^ A recent study has demonstrated that NM promotes cell survival, accelerates axonal growth, and reduces intraneural fibrosis, without any adverse effects on the nerve repair process.^[23]^ The application of safe doses of NM can also lead to a reduction in MuRf-1 protein expression by extending nerve length by up to 9%, which can reverse denervation and slow down muscle atrophy through signaling pathways.^[26]^ Numerous studies have demonstrated that glial cells, particularly astrocytes, play a significant role in central nervous system injury and that their proliferation increases following injury.^[27,28]^ Glial cells release various growth factors and cytokines that promote nerve growth and repair, as well as help to maintain the structural integrity of the Central nervous system.^[29]^ An animal study showed that NM has been shown to effectively reduce brain-derived neurotrophic factor expression in the midbrain and thalamus, as well as decrease the optical density of astrocytes and microglia, suggesting its neuroprotective effects,^[30]^ Improve body function. Overall, the mechanism of NM for nerve recovery is based on the principle of neuroplasticity, which involves increasing nerve mobility, stimulating nerve fibers, promoting nerve regeneration, increasing nerve conduction velocity, reducing pain and inflammation, and restoring normal movement patterns.^[31,32]^

### 2.2. Pain control

**Figure 1.**
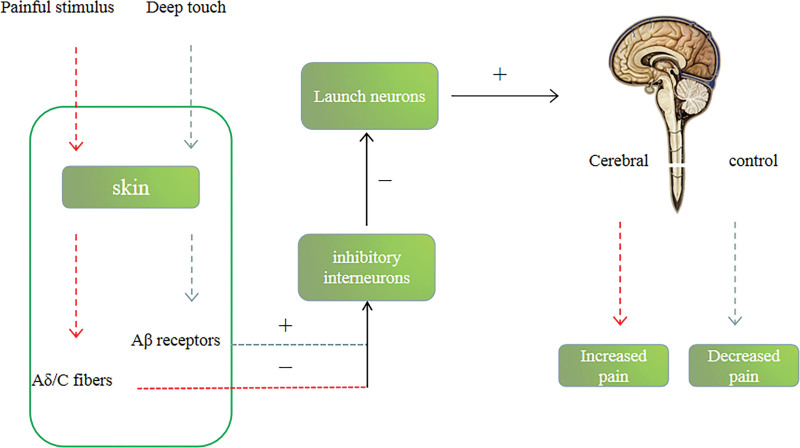
Pain gate control theory.

There is a substantial body of research that demonstrates the effectiveness of gliding techniques in NM for pain control.^[33,34]^ These techniques work through a range of mechanisms of action, including the activation of descending pain inhibitory pathways, the reduction of central sensitization, the stimulation of mechanoreceptors, and the activation of the parasympathetic nervous system.^[35–37]^ Most experts in the field agree that pain is regulated by the gate control theory, which is also supported by a diverse range of evidence, including animal studies, clinical trials, as well as physiological and biochemical experiments.^[38–40]^ The gate control theory of pain was first proposed by Melzack and Wall in 1965 and has been widely accepted by the scientific community.^[41]^ According to Melzack and Wall’s gate control theory of pain, pain signals are transmitted from the periphery to the spinal cord via fine fibers, mainly A-delta and C-fibers. These signals are then transmitted upward through the spinal cord to the brain, resulting in the perception of pain. However, the theory proposes that a coarser fiber, A-beta fibers, inhibits the transmission of pain signals by weakening the signal before it reaches the pain threshold. This closing of the “gate” prevents the information from being transmitted to the brain and results in a decrease in pain perception^[42–44]^ (Fig. 1). Currently, it is also believed that pain is controlled through the amygdala-parabrachial pathway (CeA-LPB). After injury, the parabrachial neuron activity is more intense and during pain, this inhibitory pathway is suppressed by prednisolone, growth inhibitory hormone, and adrenocorticotropin-releasing hormone. This pathway plays an important role in pain control and may become a new direction in the treatment of chronic pain in the future.^[45]^ Despite the potential involvement of these substances in pain control, it should be noted that they are also influenced by emotional and psychological factors,^[46]^ which could potentially reinforce or inhibit the pathway and affect pain facilitation. Overall, the pathways involved in pain control are highly complex and involve various regions of the brain. NM plays a crucial role in managing chronic pain by utilizing these mechanisms for pain management.

### 2.3. Inflammation control

Inflammatory changes in the lumbar vertebrae, discs, joints, muscles, fascia, nerves, and other tissues are commonly regarded as a primary contributor to the development of LBP.^[47]^ Studies have shown that patients with LBP often exhibit elevated levels of certain inflammatory markers such as interleukin-6, C-reactive protein, tumor necrosis factor-alpha, and interleukin-1β. These levels are found to be higher in patients with more severe symptoms,^[47]^ Some researchers suggest that elevated levels of interferon-γ and interleukin-10 may have anti-inflammatory effects and help to reduce inflammation.^[48]^ It is currently hypothesized that neural mobilization can help reduce the release of inflammatory substances and improve the pain status of patients.^[49]^ NM through sliding techniques can reduce the stimulation of mechanoreceptors, thereby decreasing the release of pro-inflammatory factors. However, its effectiveness is confined to the location of the manipulation.^[50]^ Some scholars have found in animal experiments that the reduction in inflammatory factors resulting from NM may be due to various factors, such as increased blood flow to the affected area and decreased afferent signal from C-fibers after the application of sliding techniques.^[50,51]^ Research has indicated that injury to body tissues can result in the blockage of blood vessels in these areas, leading to a decrease in blood flow and subsequent reduction in the levels of nitric oxide, ultimately accelerating the onset of inflammation.^[52]^ Improving blood circulation can stimulate the release of anti-inflammatory factors, such as nitric oxide, possibly by facilitating the transport of oxygen and nutrients to the affected area and removing metabolites. When the body is in pain, substances such as prostaglandin and bradykinin are released, which can accelerate the production of pro-inflammatory factors.^[53]^ When these factors accumulate to a certain amount, chronic LBP may gradually develop into other chronic diseases, such as arthritis and diabetes.^[54]^ Other scholars have proposed that the autonomic nervous system can regulate neuroinflammatory pathways via the sympathetic and parasympathetic neural pathways.^[55]^ Massage, neural mobilization, and other manipulations have been shown to effectively activate the parasympathetic nervous system and neural pathways for the effective control of inflammation. This is achieved by activating the cholinergic anti-inflammatory pathway.^[56,57]^ Overall, the exact mechanisms by which neural mobilization controls inflammation are not fully understood and likely involve multiple pathways, including the autonomic nervous system and stimulation of mechanoreceptors. Further clinical studies are needed to explore the underlying mechanisms and clinical effectiveness of NM in controlling inflammation.

## 3. Application of nerve mobilization in the treatment of low back pain

In a meta-analysis examining the effectiveness of NM therapy for LBP, it was found that there was no significant difference in the short- and medium-term resolution of LBP between the manipulative and sham groups,^[58]^ because the meta-analysis did not distinguish between the different types of manipulative therapy, it was not possible to determine whether the observed effect was specifically due to neural mobilization or not. A study conducted by Sidney M Rubinstein et al^[59]^ reported that patients who received spinal manipulation treatment had better short-term pain relief outcomes compared to other non-recommended treatments. A prospective controlled trial by Alshami AM investigated the efficacy of neural mobilization in patients with lumbar related leg pain and neurological problems. The study divided patients into 3 groups: sliding technique + transcutaneous electrical nerve stimulation (Tens), tension technique + Tens, and Tens alone. Patients received therapy 3 times a week for 2 weeks. The study found that patients who received the sliding technique and tension technique had the greatest reduction in pain during the first and third treatments, and all 3 groups showed significant improvements in joint range of motion compared to Tens alone. These results suggest that neural mobilization can effectively alleviate LBPand improve joint range of motion in the short-term.^[60]^ A clinical study reported the effectiveness of neural mobilization in reducing the Oswestry scores of a 54-year-old female patient with cauda equina root adhesion after lumbar spine surgery. The patient received neural mobilization twice a week for 3 weeks, resulting in short-term pain relief. However, since there was no postoperative follow-up, the long-term efficacy of the technique cannot be determined.^[61]^ Some scholars argue that NM may not be effective in treating nerve compression caused by LBP.^[62]^ Additionally, when soft tissue adhesion occurs, nerve roots may be relatively fixed, and neural mobilization may actually increase nerve root sensitivity, which is not the desired outcome of treatment.^[32]^ A randomized controlled trial conducted by Plaza-Manzano G provided further support for this view. The trial involved patients with lumbago, radiculalgia, and lumbar disc herniation who were divided into 2 groups: a NM + exercise control group and an exercise control group. The treatment was administered twice every 2 weeks for a total of 4 weeks, with each session lasting 30 minutes. The study found that the addition of neural mobilization to motor control significantly reduced mechanical sensitivity, but there was no significant difference in the degree of pain relief compared to the independent group.^[63]^ This may be related to factors such as the level of expertise of the therapists, the criteria used for patient selection, and the treatment plan itself. It is worth noting that most of the current clinical studies combine NM with other treatment programs, and few studies use it as a standalone treatment for LBP. The effectiveness of neural mobilization may be related to the underlying cause of LBP, as well as the skill level of the clinician and the treatment regimen employed. Furthermore, long-term efficacy studies are lacking and require validation through a larger number of clinical trials.

## 4. Discussion

NM has shown promising results in the treatment of LBP, but there are still some challenges and potential benefits to consider. One major challenge is the lack of knowledge and expertise, as it is a relatively new technique and not widely known or practiced by medical professionals. Another challenge is the lack of medical funding, as neural mobilization is not typically covered by insurance or included in hospital reimbursement lists, making it less accessible to patients. Additionally, there is a need for more research on the mechanisms and clinical efficacy of neural mobilization for LBP, as most studies to date have focused on other conditions such as carpal tunnel syndrome and radiculotic cervical spondylosis. Despite these challenges, there is potential for neural mobilization to offer a noninvasive and effective alternative to traditional LBP treatments, particularly in cases where other interventions have not been successful^[64]^; Due to the variability in patients conditions, it is difficult to determine the optimal frequency, intensity, and duration of neural mobilization due to the lack of treatment guidelines. Further research is needed to establish treatment protocols. With the advancement of technology, there are several ways to improve and monitor LBP treatment, such as the use of accelerometers. An accelerometer is a device capable of measuring various parameters such as joint angles, range of motion, and joint torque,^[65]^ and its effectiveness in monitoring the lumbar spine has been demonstrated by current research.^[66,67]^ Wearable devices have the potential to improve patient compliance and provide clinicians with more precise data during neural mobilization, allowing for the adjustment of clinical protocols. Current studies have shown that wearable devices, such as accelerometers, can effectively monitor lumbar vertebrae. In addition, virtual reality and sports games are already being utilized in biofeedback signaling, which is a novel type of feedback technology that may aid in the improvement of musculoskeletal disorders.^[68]^ Numerous studies have demonstrated that virtual reality technology can simulate various environments, alleviate patients anxiety, eliminate emotional influences, and significantly reduce the level of pain experienced by patients with low back pain.^[69,70]^ The integration of virtual technology with NM allows patients to exercise and recover in a controlled and safe environment. In summary, wearable devices, virtual reality technology, and other emerging technologies can be used to monitor and implement NM treatment for LBP, ultimately enhancing its effectiveness.

NM may have potential applications for other neurological disorders, such as neuropathic pain. Neuropathic pain is typically characterized by generalized pain resulting from lesions in the somatosensory system and represents 20% to 25% of chronic pain cases.^[71]^ Current management programs for neuropathic pain are effective, but many patients still suffer from pain that is not adequately controlled. Neuroregulatory techniques, including neural mobilization, have been identified as potential therapeutic approaches for managing this type of pain.^[72]^ NM has been shown to alleviate pain by improving neuroregulatory nerves, although there are limited research results available to support this claim, and more clinical studies are needed to prove its effectiveness. Another potential approach is to combine NM with drugs, exercise therapy, physical factor therapy, and other methods in order to learn from each other and provide the most effective treatment for LBP. Finally, the integration of knowledge from medical imaging, molecular biology, rehabilitation medicine, and other relevant disciplines can help to further explore the basic mechanisms of neural mobilization and understand how it can be used to treat different types of lumbar pain.

## 5. limitations

Only 3 databases, PubMed and Web of Science and Cochrane, were searched in this study, so it may not cover all the relevant literature, and it is hoped that in the future, researchers will be able to validate it from a wider aspect.

## Author contributions

**Investigation:** Quanzheng Chen.

**Resources:** Quanzheng Chen, Zhenshan Wang.

**Writing – original draft:** Quanzheng Chen, Zhenshan Wang.

**Writing – review & editing:** Shuna Zhang.
